# Dysphagia: Focus in Diagnosis

**DOI:** 10.1055/s-0044-1791749

**Published:** 2024-10-25

**Authors:** Geraldo Pereira Jotz

**Affiliations:** 1 Universidade Federal do Rio Grande do Sul (UFRGS), Porto Alegre, Brazil; 2 Universidade do Vale do Sinos (UNISINOS), São Leopoldo, Brazil; 3 Voice Institute, Porto Alegre, Brazil

## Diagnosis

The swallowing process is a sequence of rapid and involuntary movements in the phases after the oral phase, which conduct solid and liquid foods through the oropharynx and hypopharynx to the stomach, to nourish and hydrate the individual, ensuring their survival. 1 2 3

The World Health Organization (1995), 4 after consensus among experts, defined Quality of Life (QoL) as an individual's perception of their living conditions within the context of cultural values and systems, as well as in relation to their expectations, goals, and concerns. QoL is thus understood as the interplay of multidimensional factors based on physical, mental, and social parameters, all of which can be impacted by dysphagia. This condition can lead to social deprivation, isolation, and withdrawal from professional environments. 5

The literature presents several protocols that assess the QoL of dysphagic patients. Due to the need to differentiate individuals with normal swallowing from dysphagic individuals affected by various diseases, several protocols have been developed to detect the impact of dysphagia on QoL, such as the MD Anderson Dysphagia Inventory (MDADI), 6 the Quality of Life in Swallowing Disorders - SWAL-QOL 7 and the Dysphagia Handicap Index, 8 among others. Using these protocols is important as we seek to assess the patient. Quality of life is closely associated with the underlying disease, its repercussions, and, mainly, its management, even when we are dealing with neurodegenerative diseases and malignant neoplastic diseases where we know that even today, we have limited impact on improving the dysphagic condition of these patients, depending on the stage of the disease and the patient's response to the proposed treatment.

The change in the quality of life of patients with dysphagia is one important factor that impacts people's daily lives. Early diagnosis and management of these disorders are essential to control these symptoms.

Regarding the individual's self-assessment of their perception of dysphagia, the Eating Assessment Tool (EAT-10) is an important method of clinical analysis for detecting dysphagia, observed as difficulty in swallowing. 9

Dysphagia can present itself in isolation or with associated signs and symptoms. The evaluation of dysphagia is guided by a history of the dysphagia itself, previous medical history, eating habits, family history, use of medications, and allergies, considering the vast differential diagnosis. A complete clinical otorhinolaryngological examination and a videofibroendoscopic examination of swallowing, and testing colored foods, are essential. In this evaluation phase, the participation of a speech therapist is necessary to establish diagnostic criteria and plan the shared medical-speech therapist treatment model. The involvement of other medical professionals, such as neurologists and gastroenterologists, is sometimes required, as well as nutritionists and psychologists, among others.

Essentially, cranial nerves are components of the peripheral nervous system that connect to the brain and not to the spinal cord. Twelve pairs of cranial nerves can be found on the brain's surface, each with a name related to its aspect or function. Regarding swallowing, the participation of five cranial nerves in this process is already consolidated in the literature. They are V (fifth), VII (seventh), IX (ninth), X (tenth), and XII. Some of the cranial nerves transmit afferent information from the sensory organs, while other nerves send efferent signals to effector organs, such as muscles and glands, and others have both afferent and efferent fibers, therefore, they are mixed nerves. The importance of studying cranial nerves is based on the fact that these delicate structures are fundamental for basic functions of the human body, in addition to swallowing, such as smell, vision, taste, hearing, balance, pain, speech, and blood pressure control.

Testing these cranial nerves in otorhinolaryngological care is easy to perform and has a vital clinical significance for diagnostic elucidation. It should be part of the otorhinolaryngological routine. As for the videofibroendoscopic examination, the joint evaluation by the otorhinolaryngologist and the speech therapist, testing different colored consistencies, suggests the use of a food thickener in the water, using different consistencies, using blue food coloring which, in our experience, is what provides the best contrast. For testing the liquid, without thickener, blue food coloring can be used or, exceptionally, green food coloring, when there is doubt about what may be causing laryngotracheal penetration or aspiration.

Differential diagnoses include neurological diseases, gastrointestinal diseases, metabolic diseases, infectious diseases, inflammatory diseases, iatrogenic causes, autoimmune diseases, psychiatric diseases, and side effects of certain medications, among other causes.

G *lobus faríngeus* has been associated with structural lesions of the oropharynx, upper esophageal sphincter disorders, esophageal disorders, gastroesophageal reflux disease, psychosocial factors, and psychiatric comorbidities. However, results are often contradictory, and literature remains highly inconclusive. 10

The diagnostic methods are the most diverse, from swallowing videofibroendoscopy, videofluoroscopy, 24-hour esophageal pH monitoring, manometry, esophagoscopy, FEEST (fiberoptic endoscopic evaluation of swallowing with sensory testing), Magnetic Resonance Imaging, Computerized Tomography, Scintigraphy, and PET Scan.

In this scenario, and with increasing survival rates and an aging population, swallowing disorders and their role in causing pulmonary and nutritional pathologies are becoming extremely important. Over the last two decades, the study of oropharyngeal dysphagia has been approached from various disciplines, with considerable progress in understanding its pathophysiology. 11

We consider that the patient to be evaluated came to the first appointment without prior investigation of the topic because, in the cases of dysphagic patients, particularity is the rule, and the detail makes the difference in the diagnostic outcome.

It is observed that there are literature reports where the incidence of risk for swallowing disorders can reach 37.27% in healthy people over 60 years of age. 12 13

A study conducted by Dall'Oglio et al. demonstrated that age does not have a linear influence on swallowing in healthy adults and the elderly. However, individuals aged ≥80 years had a higher prevalence of residues, and individuals aged ≥60 years had a higher prevalence of salivary stasis, suggesting a higher risk or presence of dysphagia. 14

## Conclusion

Given the above, we believe that training medical professionals to act in the early diagnosis of the disease that causes the dysphagic symptom and, together with speech-language pathologists, to plan the treatment and monitoring of patients, which only increases as the population ages, is of utmost importance for controlling the deleterious effects of the symptom and the underlying disease that causes dysphagic conditions.
